# Prognostic value of triglyceride-glucose index in patients with cardiovascular-kidney-metabolic syndrome undergoing percutaneous coronary intervention

**DOI:** 10.3389/fcvm.2025.1687231

**Published:** 2026-01-12

**Authors:** Caimei Yao, Huanting Liu, Youcheng Wang, Ziyun Wen, Yongxin Huang, Lichan Ren, Chao An, Xiyuan Mo, Jiahong Chen, Qiushuang Lin, Genghao Lu, Yimeng Yin, Liqiu Yan

**Affiliations:** 1 Department of Cardiology, The Affiliated Dongguan Songshan Lake Central Hospital, Guangdong Medical University, Dongguan, Guangdong, China; 2 Dongguan Key Laboratory of Cardiovascular Aging and Myocardial Regeneration, Dongguan Cardiovascular Institute, Dongguan, China; 3 Department of Cardiology, Dongguan Chashan Hospital, Dongguan, China; 4 Department of Medicine, Dongguan Dongkeng Hospital, Dongguan, China; 5 Department of Medicine, Dongguan Huangjiang Hospital, Dongguan, China; 6 Department of Cardiology, Dongguan Qingxi Hospital, Dongguan, China; 7 School of Public Health, Guangdong Medical University, Dongguan, China

**Keywords:** all-cause mortality, cardiac mortality, cardiovascular-kidney-metabolic syndrome, insulin resistance, triglyceride-glucose index

## Abstract

**Background:**

The triglyceride-glucose (TyG) index demonstrates strong links to heightened cardiovascular risk and progressive renal dysfunction. However, its prognostic implications in individuals diagnosed with cardiovascular-kidney-metabolic (CKM) syndrome who underwent Percutaneous Coronary Intervention (PCI) have yet to be fully elucidated.

**Methods:**

A retrospective investigation was conducted involving CKM patients who underwent PCI between January 2014 and September 2017. The TyG index categories were established utilizing X-tile software for classification purposes. The principal endpoints comprised 5-year all-cause mortality (ACM) and cardiac mortality (CM). Associations between the TyG index and ACM/CM were evaluated using Cox proportional hazards models, and further examined through restricted cubic spline (RCS) analyses.

**Results:**

Of the 2,040 patients analyzed, 1,186 (58.14%) were male and 854 (41.86%) female, with 55.83% aged ≥65 years. After analysis with multivariate Cox regression, elevated TyG index measurements demonstrated a notable association with heightened probabilities of ACM and CM occurrence. In comparison to the medium TyG index group, individuals categorized within the low TyG index group exhibited markedly elevated risks for ACM [hazard ratio [HR] = 1.82, 95% confidence interval [95%CI]: 1.15–2.88] and CM (HR = 2.68, 95%CI: 1.32–5.43). Additionally, a higher ACM risk was noted in the high TyG index group (HR = 1.39, 95%CI: 1.01–1.92). The RCS analysis identified no nonlinear association between the TyG index and either outcome (*P*-values for nonlinearity test: 0.177 and 0.153, respectively).

**Conclusions:**

The TyG index independently predicted increased risks of all-cause and cardiac mortality, thus highlighting its utility for risk stratification in CKM syndrome patients following PCI.

## 1 Introduction

Coronary artery disease (CAD) and chronic kidney disease (CKD) represent substantial health challenges worldwide, substantially impacting global disability and mortality rates (1, 2). Epidemiological evidence has revealed that renal impairment of varying severity is commonly observed in individuals diagnosed with CAD, while cardiovascular disease (CVD) remains the leading factor in both illness and death among individuals with CKD (3). Percutaneous coronary intervention (PCI) stands as an essential treatment approach for CAD, aimed at alleviating coronary artery stenosis or occlusion and enhancing myocardial perfusion (4). Nevertheless, clinical findings have indicated that, despite technically successful PCI, considerable residual cardiovascular risk persists, as reflected in the increased incidence of major adverse cardiovascular events (MACE) and all-cause mortality (ACM) (5–7).

In recent years, cardiovascular-kidney-metabolic (CKM) syndrome has garnered increasing scholarly interest owing to the intricate pathophysiological interplay among cardiac, renal, and metabolic dysfunctions. Numerous investigations indicate that insulin resistance (IR) constitutes a fundamental pathological nexus linking cardiovascular complications with renal functional decline (8–12). Patients diagnosed with CKM syndrome commonly exhibit a constellation of IR, persistent low-grade inflammation, and heightened oxidative stress, collectively expediting the advancement of atherosclerosis and potentially exerting a profound influence on clinical prognosis. The triglyceride-glucose (TyG) index, computed from fasting triglycerides (TG) and fasting plasma glucose, has been recognized as a straightforward yet robust proxy for IR, and has been repeatedly correlated with the onset, exacerbation, and unfavorable outcomes of CVD. Recent clinical findings have indicated that a heightened TyG index independently forecasts coronary artery disease severity and MACE occurrence among general and diabetic cohorts alike (13). Nevertheless, its predictive validity for long-term prognosis following PCI remains inadequately characterized in this distinct, high-risk group presenting simultaneous cardiac, renal, and metabolic impairments. Hence, this investigation sought to examine the prognostic significance of the TyG index regarding outcomes in CKM patients receiving PCI treatment.

## 2 Methodologies and materials

### 2.1 Research methodology and participants

**Figure 1:**
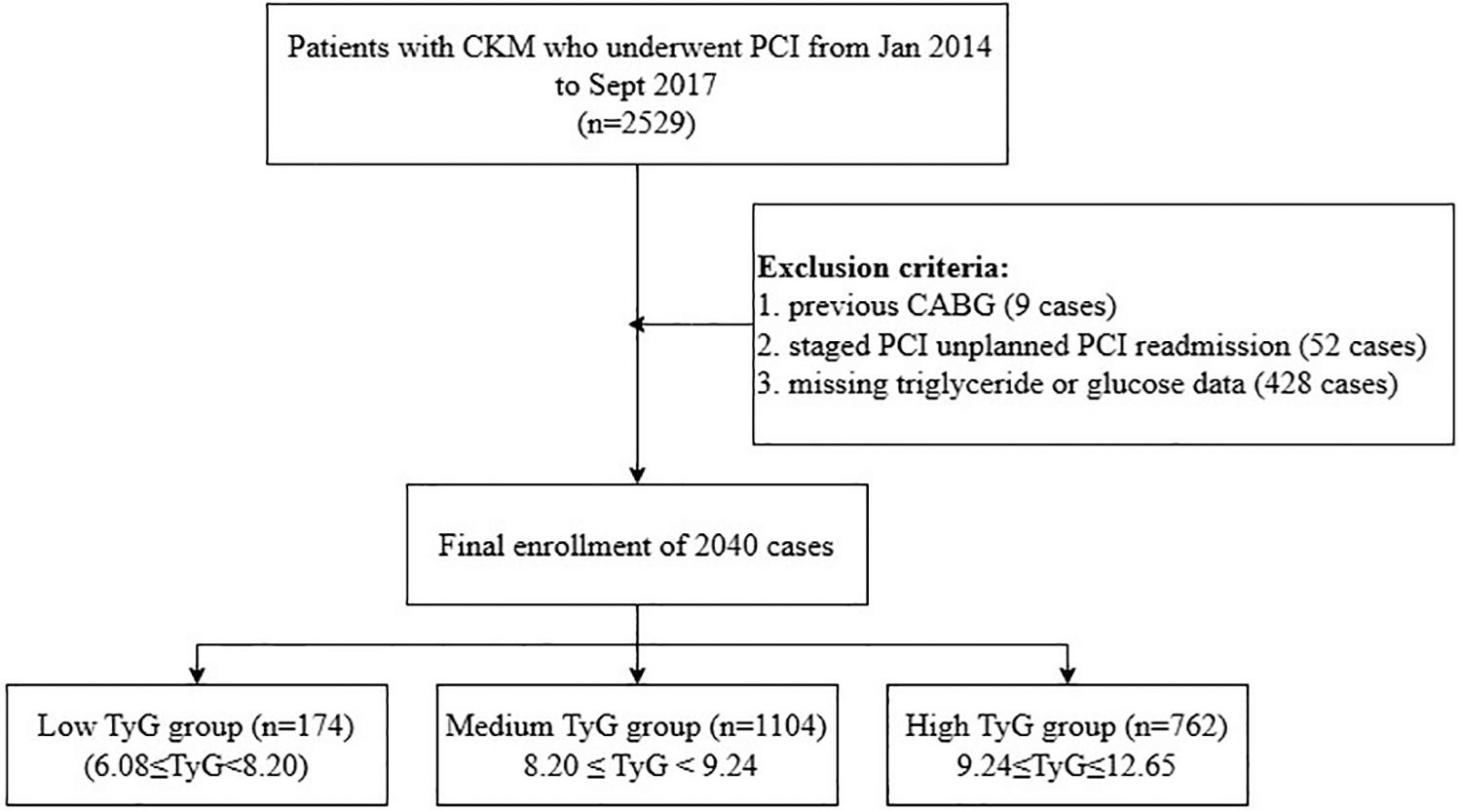
Study flowchart.

In this retrospective analysis, a sum of 2,529 CKM patients receiving PCI treatment at Cangzhou Central Hospital from January 2014 through September 2017 underwent initial evaluation. The selection standards included: (1) individuals aged 18 years or above; (2) confirmed CAD requiring PCI intervention; and (3) calculated glomerular filtration rate below 90 mL/min/1.73 m². Subjects were not eligible if they presented any of these factors: (1) history of coronary artery bypass grafting; (2) staged or unplanned PCI readmission; or (3) incomplete TG or glucose data. Following the application of these criteria, 2,040 individuals were ultimately included in the analysis (Figure 1).

### 2.2 Data collection and definitions

The data utilized for analytical purposes in this study were procured from medical records, encompassing demographic characteristics, smoking status, comorbid conditions, and other relevant baseline parameters. Fasting venous blood specimens were procured and subsequently examined in the clinical laboratory to determine levels of low-density lipoprotein cholesterol (LDL-C), TG, high-density lipoprotein cholesterol (HDL-C), total cholesterol (TC), and fasting blood glucose (FBG).

The TyG index was computed based on the following formula (14, 15):

$$TyG=Ln\left(\frac{TG(mg/dL)\times FBG(mg/dL)}{2}\right)$$

ACM was stratified based on TyG index thresholds, which had been determined through predictive modeling using X-tile software. The cohort was split into three categories: low TyG group (6.08 ≤ TyG < 8.20, *n* = 174), medium TyG group (8.20 ≤ TyG < 9.24, *n* = 1,104), and high TyG group (9.24 ≤ TyG ≤ 12.65, *n* = 762).

Ethical approval for this investigation was sanctioned by the Ethics Committee of Cangzhou Center, affiliated with Hebei Medical University. Each participant completed consent documentation.

### 2.3 Statistical analysis

Regarding continuous variables, central tendency and dispersion were denoted as mean ± standard deviation when data exhibited normal distribution, while non-normally distributed parameters were represented through median and interquartile range. Group comparisons utilized one-way ANOVA for normally distributed measurements, whereas the Kruskal–Wallis test analyzed non-normally distributed data. Categorical parameters were displayed as frequencies (percentages), with analyses performed via chi-square testing. To preliminarily identify variables potentially associated with clinical outcomes, univariate Cox regression was executed. To minimize the risk of Type II error associated with stringent thresholds, a relaxed criterion (*P* < 0.10) was adopted for initial variable screening. Those found to be significant were subsequently incorporated into the multivariate Cox model with TyG, yielding hazard ratios (HRs) and 95% confidence intervals (CIs). Kaplan–Meier methodology was applied to construct cumulative survival curves. To examine possible nonlinear links between the TyG index and ACM, restricted cubic spline (RCS) regression was implemented. Subgroup analyses were carried out to assess variations in TyG index effects across populations, stratified by the following factors: sex (male, female), hypertension (yes, no), diabetes (yes, no), prior MI (yes, no), prior PCI (yes, no), and previous stroke (yes, no). The handling of missing data was performed as follows. Patients with missing values for the key variables required to calculate the TyG index (fasting triglycerides or fasting glucose) were excluded from the initial cohort selection, as detailed in Figure 1. For the remaining patients included in the final analysis (*n* = 2,040), the completeness of data for other covariates was high. Continuous variables were imputed using predictive mean matching, and categorical variables were imputed with the mode (the most frequent category).

All statistical evaluations were executed utilizing R software (v4.4.1), with statistical significance established at a two-sided *P* < 0.05.

## 3 Results

### 3.1 Baseline characteristics

Table 1 presents the baseline clinical characteristics of CKM patients who underwent PCI. Upon stratification by TyG index tertiles, statistically significant variations in age and sex distribution were identified across the subgroups (*P* < 0.001). The age composition was as follows: 18–45 years (2.25%), 45–65 years (41.91%), and ≥65 years (55.83%), with a greater proportion of males compared to females (58.14% vs. 41.86%). Notably, the prevalence of cardiovascular risk factors (CRFs) exhibited a progressive increase with rising TyG levels, particularly for diabetes mellitus (48.3% vs. 32.6% vs. 19.1%, *P* < 0.001) and hypertension (82.4% vs. 76.8% vs. 69.2%, *P* < 0.001). These patterns indicate that elevated TyG indices are linked to a more adverse CRF.

**Table 1:** Baseline characteristics of patients receiving PCI who have CKM.

Valuables	Total (*n* = 2,040)	Low TyG (*n* = 174)	Medium TyG (*n* = 1,104)	High TyG (*n* = 762)	*P*
		6.08 ≤ TyG < 8.20	8.20 ≤ TyG < 9.24	9.24 ≤ TyG ≤ 12.65	
Age					
<45	46 (2.25%)	4 (2.30%)	20 (1.81%)	22 (2.89%)	<0.001
45∼65	855 (41.91%)	53 (30.46%)	435 (39.40%)	367 (48.16%)	
≥65	1,139 (55.83%)	117 (67.24%)	649 (58.79%)	373 (48.95%)	
Gender					
Male	1,186 (58.14%)	126 (72.41%)	656 (59.42%)	404 (53.02%)	<0.001
Female	854 (41.86%)	48 (27.59%)	448 (40.58%)	358 (46.98%)	
Diabetes					
No	1,564 (76.67%)	160 (91.95%)	957 (86.68%)	447 (58.66%)	<0.001
Yes	476 (23.33%)	14 (8.05%)	147 (13.32%)	315 (41.34%)	
Hypertension					
No	648 (31.76%)	71 (40.80%)	372 (33.70%)	205 (26.90%)	<0.001
Yes	1,392 (68.24%)	103 (59.20%)	732 (66.30%)	557 (73.10%)	
COPD					
No	2,011 (98.58%)	172 (98.85%)	1,085 (98.28%)	754 (98.95%)	0.461
Yes	29 (1.42%)	2 (1.15%)	19 (1.72%)	8 (1.05%)	
Previous PCI					
No	1,780 (87.25%)	157 (90.23%)	955 (86.50%)	668 (87.66%)	0.357
Yes	260 (12.75%)	17 (9.77%)	149 (13.50%)	94 (12.34%)	
Previous MI					
No	1,885 (92.40%)	161 (92.53%)	1,015 (91.94%)	709 (93.04%)	0.674
Yes	155 (7.60%)	13 (7.47%)	89 (8.06%)	53 (6.96%)	
Previous stroke					
No	1,816 (89.02%)	159 (91.38%)	975 (88.32%)	682 (89.50%)	0.421
Yes	224 (10.98%)	15 (8.62%)	129 (11.68%)	80 (10.50%)	
Smoking					
No	1,802 (88.33%)	154 (88.51%)	964 (87.32%)	684 (89.76%	0.270
Yes	238 (11.67%)	20 (11.49%)	140 (12.68%)	78 (10.24%)	
Systolic pressure	131.00 (122.00–147.00)	133.50 (126.00–150.00)	132.00 (124.00–147.00)	130.00 (120.00–147.00)	0.193
Diastolic pressure	80.00 (70.00–88.00)	80.00 (70.00–89.75)	80.00 (70.00–87.00)	80.00 (70.00–89.00)	0.914
Total cholesterol	4.40 (3.75–5.10)	3.90 (3.30–4.60)^a,b^	4.21 (3.70–4.90)^b^	4.60 (4.00–5.40)	<0.001
Hemoglobin	132.00 (122.00–142.00)	131.83 (122.00–139.75)	132.00 (122.00–142.00)	132.00 (122.00–143.00)	0.138
ALT	22.70 (15.00–39.05)	18.05 (12.25–32.60)^a,b^	21.45 (14.60–37.24)^b^	25.25 (16.05–43.88)	<0.001
AST	23.00 (17.40–46.32)	23.00 (17.00–39.17)	22.30 (17.00–45.08)	23.75 (18.00–50.25)	0.071
HDL-C	0.93 (0.79–1.08)	1.04 (0.91–1.26)^a,b^	0.96 (0.82–1.10)^b^	0.87 (0.74–1.01)	<0.001
LDL-C	2.51 (2.05–3.10)	2.27 (1.78–2.76)^a,b^	2.52 (2.05–3.11)	2.59 (2.09–3.12)	<0.001

COPD, chronic obstructive pulmonary disease; MI, myocardial infarction; ALT, alanine aminotransferase; AST, aspartate aminotransferase.

a,b Indicate statistically significant differences from the medium TyG group and high TyG group, respectively.

### 3.2 Link between TyG index and CRFs

Correlation analyses utilizing Spearman and Pearson methods were executed to evaluate the links between the TyG index and various CRFs. As depicted in Table 2, the TyG index exhibited positive links to Hemoglobin, ALT, AST, and LDL-C, whereas negative associations were identified with age, TC, and HDL-C.

**Table 2:** Link between the TyG index and cardiovascular risk factors.

Valuables	Correlation coefficient (r)	*P* value
Age	−0.135	<0.001
Systolic pressure	−0.031	0.157
Diastolic pressure	−0.013	0.547
Total cholesterol	−0.135	<0.001
Hemoglobin	0.050	0.025
ALT	0.051	0.020
AST	0.045	0.044
HDL-C	−0.280	<0.001
LDL-C	0.088	<0.001

### 3.3 Univariate cox regression analysis

Univariate Cox regression analysis identified several variables markedly linked to ACM, as depicted in Supplementary Table 1. These included: age ≥65 years (HR = 2.42, 95%CI: 1.66–3.52, *P* < 0.001), chronic obstructive pulmonary disease (COPD) (HR = 3.79, 95%CI: 1.78–8.10, *P* < 0.001), alanine aminotransferase (ALT) (HR = 1.002, 95%CI: 1.001–1.003, *P* < 0.001), and aspartate aminotransferase (AST) (HR = 1.002, 95%CI: 1.001–1.003, *P* < 0.001).

Moreover, as indicated in Supplementary Table 2, age ≥65 years (HR = 2.42, 95%CI: 1.33–4.42, *P* = 0.004) and individuals with COPD (HR = 4.15, 95%CI: 1.30–13.24, *P* = 0.016) emerged as independent predictors of cardiac mortality (CM).

### 3.4 Link between TyG index and outcome

**Figure 2:**
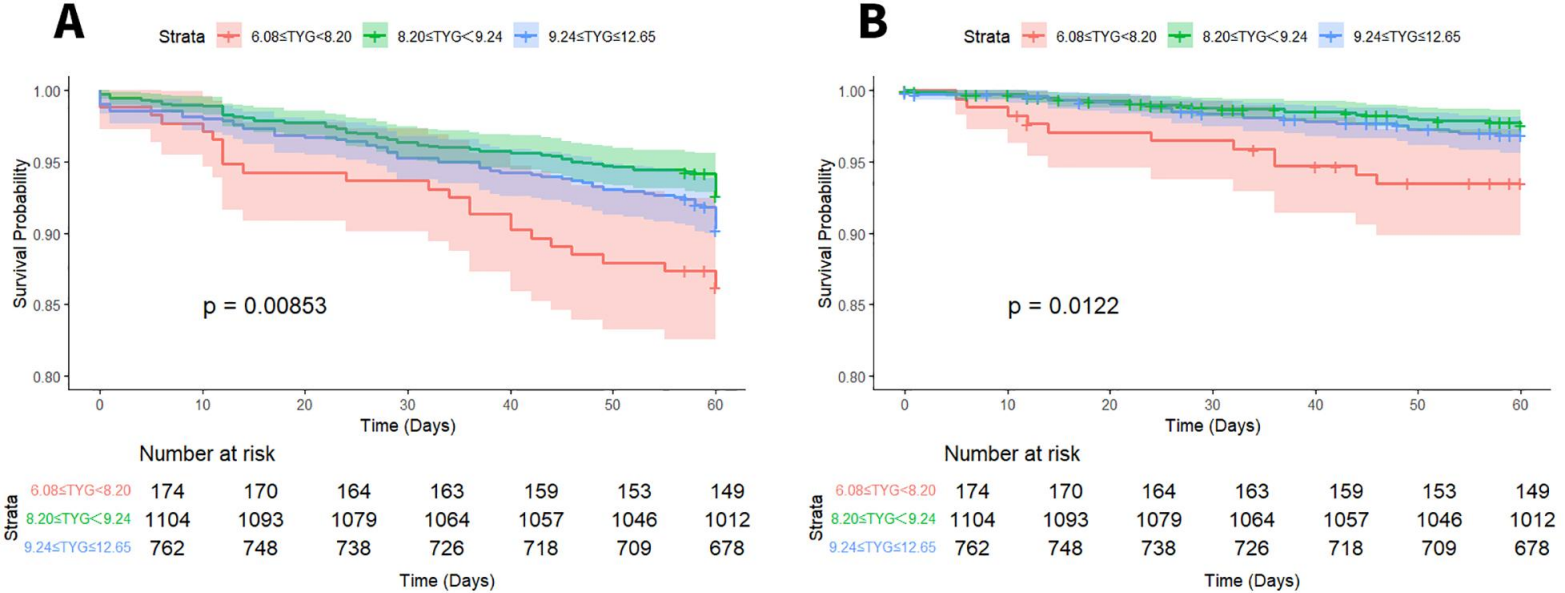
Kaplan–Meier survival curves for ACM **(A)** and CM **(B)** based on TyG index stratification.

Long-term survival outcomes were assessed through Kaplan–Meier curve analysis. Statistically significant variations in 5-year ACM were identified across the three groups stratified by TyG index (log-rank *P* < 0.001). Specifically, comparisons between the low-TyG and medium-TyG groups revealed notable differences in both ACM (log-rank *P* = 0.008) and CM (log-rank *P* = 0.012), as illustrated in Figure 2.

Multivariate Cox regression models were applied to examine the links between TyG index levels and both ACM and CM in CKM patients undergoing PCI. Details of the unadjusted and adjusted models are depicted in Table 3. For ACM, in the unadjusted Model 1, the low-TyG group (6.08 ≤ TyG < 8.20) exhibited a markedly elevated risk of ACM vs. the medium-TyG group (8.20 ≤ TyG < 9.24) (HR = 1.96, 95%CI: 1.25–3.10; *P* < 0.05). After controlling for age, increased risks remained evident in both the low-TyG and high-TyG groups (TyG ≥ 9.24), with HRs of 1.85 (95%CI: 1.17–2.91) and 1.46 (95%CI: 1.06–2.00), respectively (all *P* < 0.05). Further adjustment in Model 3 for age, COPD, AST, ALT, and hemoglobin preserved the statistical significance of the associations (low-TyG: HR = 1.88, 95%CI: 1.19–2.97; high-TyG: HR = 1.37, 95%CI: 1.01–1.89; all *P* < 0.05). Regarding CM, the low-TyG group consistently exhibited an elevated risk across all models: unadjusted (HR = 2.80, 95%CI: 1.38–5.67), age-adjusted (HR = 2.65, 95%CI: 1.31–5.38), and model 3 adjusted (HR = 2.75, 95%CI: 1.35–5.28) (all *P* < 0.05).

**Table 3:** Findings from multivariate Cox regression assessing the link between the TyG index and endpoints.

Group	Model 1		Model 2		Model 3	
	HR (95% CI)	*P*	HR (95% CI)	*P*	HR (95% CI)	*P*
ACM						
6.08 ≤ TyG < 8.20	1.96 (1.25,3.10)	0.004	1.85 (1.17,2.91)	0.008	1.88 (1.19,2.97)	0.007
8.20 ≤ TyG < 9.24	Reference		Reference		Reference	
9.24 ≤ TyG ≤ 12.65	1.34 (0.98,1.84)	0.067	1.46 (1.06,2.00)	0.020	1.37 (1.01,1.89)	0.05
CM						
6.08 ≤ TyG < 8.20	2.80 (1.38,5.67)	0.004	2.65 (1.31,5.38)	0.007	2.75 (1.35,5.58)	0.005
8.20 ≤ TyG < 9.24	Reference		Reference		Reference	
9.24 ≤ TyG ≤ 12.65	1.30 (0.74,2.27)	0.36	1.38 (0.79,2.43)	0.25	1.38 (0.78,2.43)	0.267

ACM: Model 1: no adjustment; Model 2: adjustment for age; Model 3: adjustment for age, COPD, AST, ALT, SYNTAX Score. CM: model 1: no adjustment; model 2: adjustment for age; model 3: adjustment for age, COPD.

**Figure 3:**
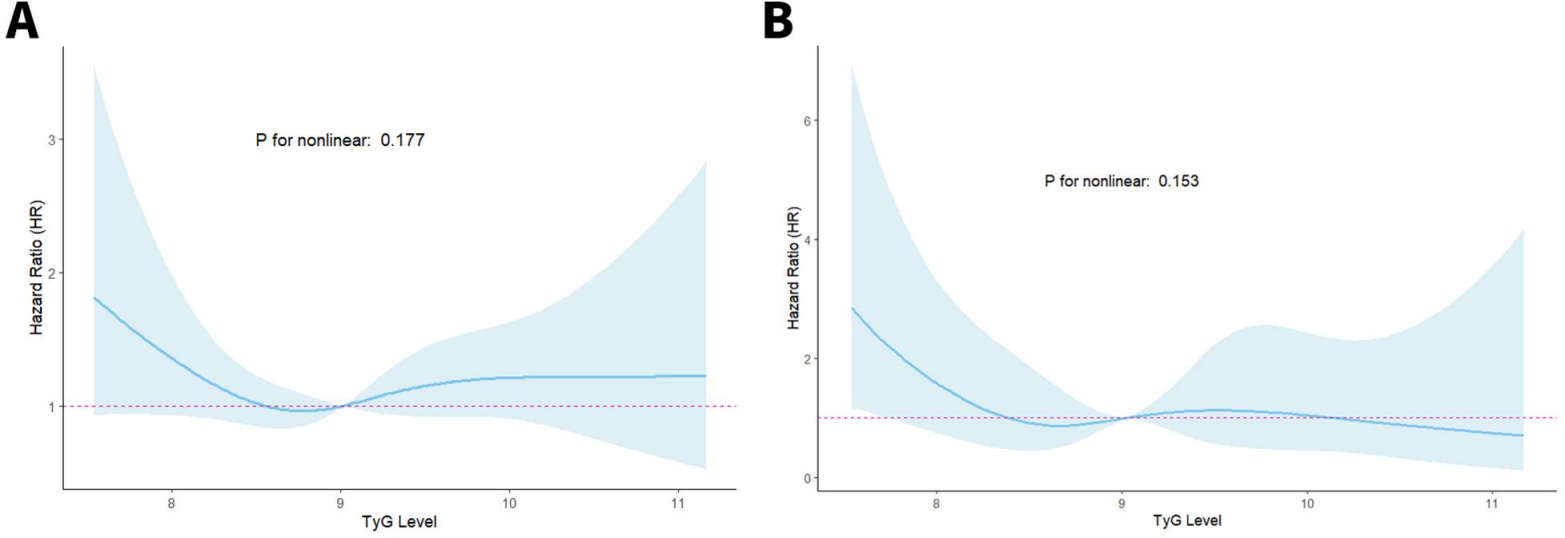
RCS curves of the TyG index connected to ACM **(A)** and CM **(B)** risks.

TyG index and ACM correlation patterns in CKM patients after PCI were assessed through RCS regression analysis to examine potential nonlinear associations. As illustrated in Figure 3, no evidence of a nonlinear link was identified between the TyG index and either ACM or CM (*P* for nonlinearity > 0.05 for all comparisons).

### 3.5 Subgroup analysis

Subgroup stratification analyses were executed to systematically investigate the link between TyG index levels and both ACM and CM across varying clinical profiles. Patients were categorized according to sex, history of hypertension, diabetes mellitus, previous myocardial infarction, prior PCI, and prior stroke.

**Figure 4:**
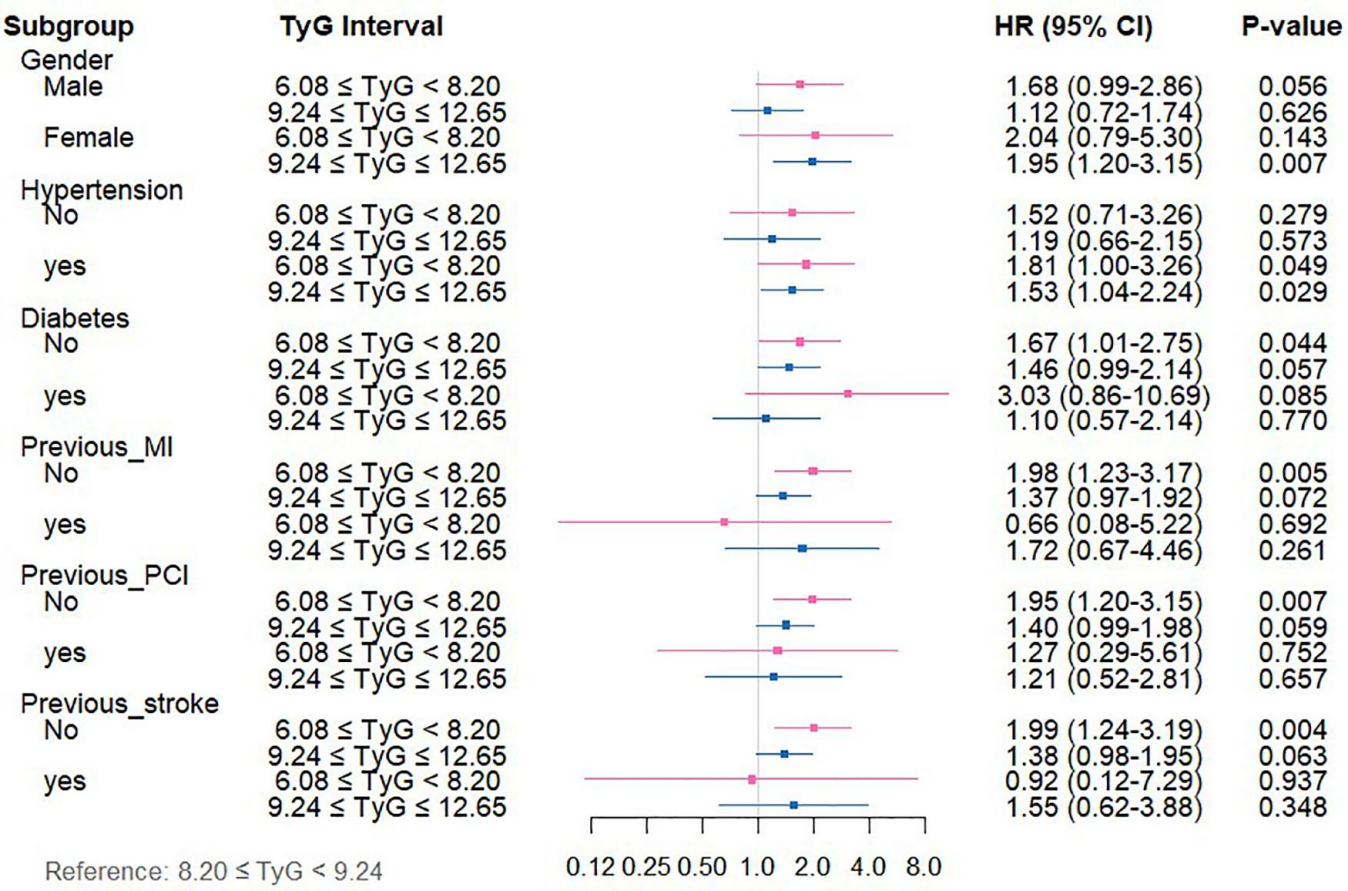
Forest plot depicting TyG index measurements and ACM risk across distinct patient subgroups.

Regarding ACM (Supplementary Table 3, Figure 4), among patients in the low-TyG group, markedly elevated ACM risks emerged in specific subsets vs. the medium-TyG group (all *P* < 0.05): individuals experiencing hypertension (HR = 1.81, 95%CI: 1.00–3.26), subjects lacking diabetes (HR = 1.67, 95%CI: 1.01–2.75), individuals without MI history (HR = 1.98, 95%CI: 1.23–3.17), patients with no previous PCI (HR = 1.95, 95%CI: 1.20–3.15), and individuals lacking stroke history (HR = 1.99, 95%CI: 1.24–3.19). The high-TyG group demonstrated elevated ACM risks among female subjects (HR = 1.95, 95%CI: 1.20–3.15) and individuals with hypertension (HR = 1.53, 95%CI: 1.04–2.24) (all *P* < 0.05).

**Figure 5:**
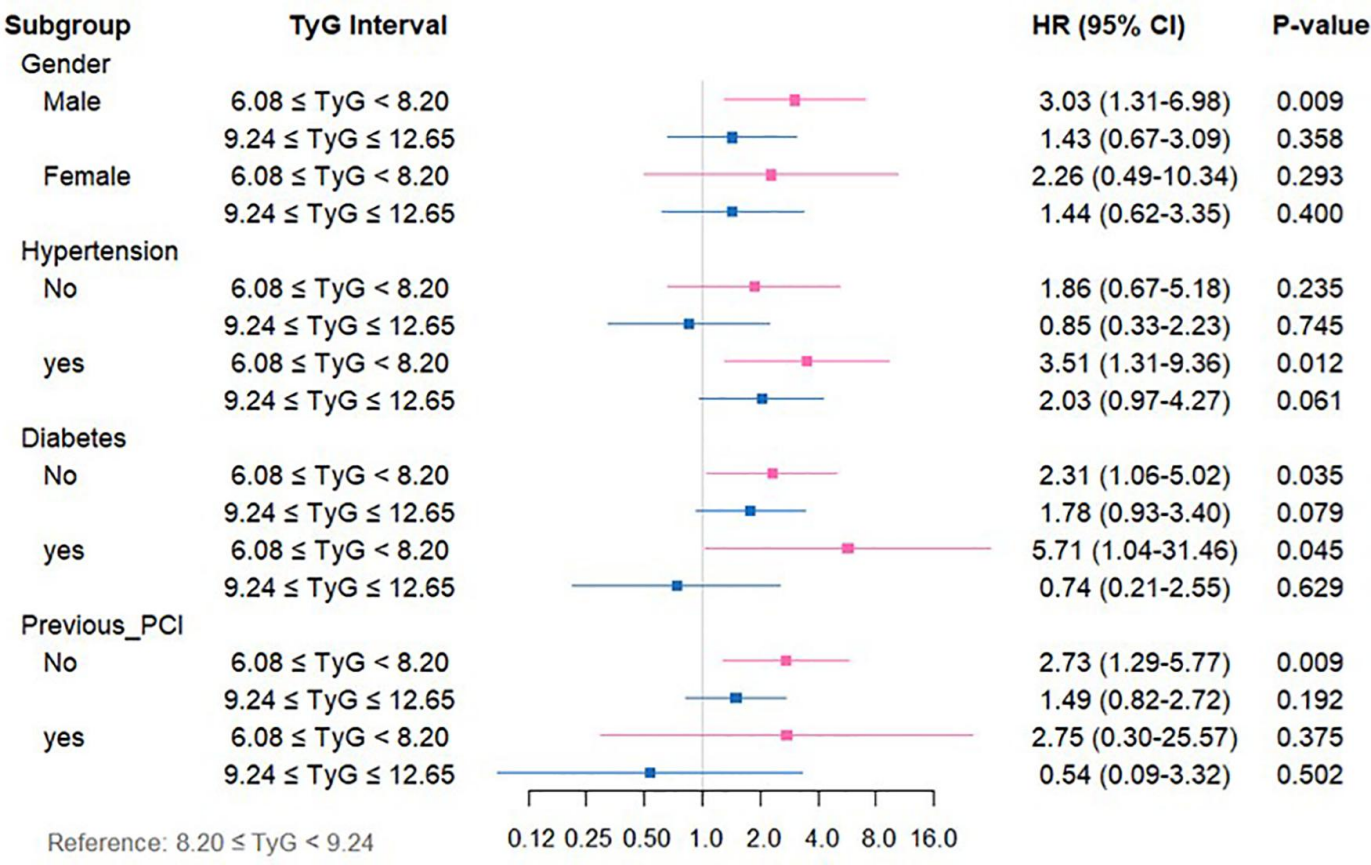
Forest plot depicting TyG index measurements and CM risk across distinct patient subgroups.

For CM (Supplementary Table 4, Figure 5), relative to the medium-TyG group, the low-TyG group exhibited markedly higher CM risks in the following subgroups (all *P* < 0.05): males (HR = 3.03, 95%CI: 1.31–6.98), individuals with hypertension (HR = 3.51, 95%CI: 1.31–9.36), those with diabetes (HR = 5.71, 95%CI: 1.04–31.746), those without diabetes (HR = 2.31, 95%CI: 1.06–5.02), and subjects without previous PCI (HR = 2.73, 95%CI: 1.29–5.77).

## 4 Discussion

This study represents the first to specifically target CKM patients as the research cohort and to report five-year follow-up outcomes, thereby offering clinically stable and reliable guidance. Moreover, the TyG index exhibits considerable value as a predictive marker in CKM patients, attributable to its ease of calculation, reproducibility, and cost-effectiveness. Although considerable evidence exists linking the TyG index to CVD outcomes, its prognostic relevance in those with CKM remains insufficiently investigated. In this study comprising 2,040 individuals with CKM, the following findings were observed: (1) The TyG index, recognized as a dependable surrogate for IR, was markedly linked to established CAD risk factors, aligning with prior research; (2) TyG levels were associated with both ACM and CM. Specifically, the low-TyG group exhibited markedly elevated risks of ACM and CM vs. the medium-TyG group, whereas the high-TyG group was independently linked to elevated ACM risk; (3) The associations between TyG levels and ACM/CM were particularly pronounced within certain clinical subpopulations, such as individuals with a history of hypertension, absence of diabetes, and no previous PCI.

CKM is recognized as a multisystem disorder arising from intricate pathophysiological interactions among metabolic disturbances (e.g., obesity and IR), CKD, and CVD, ultimately contributing to systemic multi-organ impairment. In this framework, IR, established as a key factor in atherosclerosis development, demonstrates notable correlation with both CAD initiation and advancement (16). The TyG index functions as a reliable and uncomplicated proxy indicator for IR (17). Recent medical findings indicate that TyG index values are linked to unfavorable cardiovascular results, encompassing coronary heart disease and cardiac failure. Research performed by Su et al. examining 731 coronary heart disease patients demonstrated that TyG index measurements corresponded to CAD intensity and successfully detected patients facing elevated risks of multivessel disease (18). Additionally, a forward-looking cohort investigation by Zhang et al. (*N* = 1,932) examining individuals with acute myocardial infarction and type 2 diabetes determined that higher TyG index measurements predicted elevated CM and ACM risk levels (19).

This study represents the first to establish that elevated and diminished TyG indices independently correlate with increased mortality risk among individuals with CKM, a finding that markedly contrasts with the conclusions of most earlier studies. Although numerous investigations (20–22) have identified a monotonic positive connection between the TyG index and cardiovascular events, such analyses have predominantly concentrated on general cardiovascular populations. The observed divergence may be attributable to the distinct multisystem pathological interplay present in CKM patients. Specifically, the increased risk associated with high TyG levels is consistent with classical IR theory, which facilitates the development of atherosclerosis and heart failure via disruptions in lipid and glucose metabolism (23). In contrast, the mechanism underlying the heightened risk linked to low TyG levels has been supported by several studies, including a retrospective analysis involving 19,420 participants, which reported a nonlinear relationship between TyG index and ACM or CM (24). Preliminary indications of such a nonlinear trend have also been detected in patients with metabolic diseases coexisting with CKD. For instance, the study by Xia et al. identified an inverse correlation between TG levels and mortality risk in individuals with CAD, introducing the concept of the “TG paradox” (25). Particularly noteworthy is the recognition of distinct TyG risk patterns in populations with renal insufficiency and metabolic dysregulation. Prior research identified a nonlinear (J-shaped) link between TyG index and CKD among subjects with impaired glucose metabolism and hypertension (26), while findings from Shang et al. (27) uncovered a U-shaped pattern between TyG index and disease prevalence in diabetic nephropathy, suggesting that synergistic injury involving the cardiovascular-kidney-metabolic axis may intensify the pathological consequences of extreme TyG values. Collectively, these findings imply that in cardiovascular populations with concurrent renal dysfunction and metabolic abnormalities, the risk trajectory of the TyG index may diverge from conventional linear interpretations. The elevated risk in the low TyG group, while unexpected, may reflect a state of “metabolic frailty” in advanced CKM syndrome. In these patients, low triglyceride and glucose levels could indicate poor nutritional status and chronic illness severity rather than metabolic health. This aligns with the “obesity paradox” seen in other chronic diseases, where traditional risk factors reverse direction (28, 29).

## 5 Limitations

This study has several limitations that should be carefully considered when interpreting the findings. Firstly, this was a single-center, retrospective study, which may introduce selection bias and limit the generalizability of our findings. Future multi-center prospective studies are needed to validate these results. Second, although we performed extensive subgroup analyses to explore the consistency of the TyG index's prognostic value, these analyses were exploratory and were not adjusted for multiple comparisons. Therefore, the results should be interpreted with caution, as the risk of Type I errors (false-positive findings) is increased. Some subgroups also had relatively small sample sizes, which may limit the statistical power and increase the risk of Type II errors. A further limitation pertains to the use of X-tile software for determining the TyG index cut-off values. While this data-driven approach objectively identifies thresholds that best separate groups based on the outcome (all-cause mortality), it may optimize the association specifically within our dataset, potentially leading to overfitting and an overestimation of the true effect sizes. Therefore, the prognostic utility of these specific cut-off values requires validation in future independent cohorts. Finally, the relatively small sample size in the low TyG group (*n* = 174) may limit the statistical stability of its risk estimates, and these findings require validation in larger cohorts. In addition, our analysis could not adjust for certain potential confounders, including BMI, medication use (e.g., statins, antidiabetic drugs), and albuminuria, due to data limitations.

## 6 Conclusion

TyG index is markedly linked to adverse clinical outcomes in patients with CKM, and this association persists even after adjustment for multiple confounding variables. Therefore, in managing CKM patients, clinicians should not only intensify control of conventional CRFs but also consider close monitoring of the TyG index. Nonetheless, whether modification of TyG index levels can effectively reduce mortality risk remains to be determined and warrants verification through prospective interventional studies.

## Data Availability

The raw data supporting the conclusions of this article will be made available by the authors, without undue reservation.
