# Supplementary material for: Prognostic value of triglyceride-glucose index in patients with cardiovascular-kidney-metabolic syndrome undergoing percutaneous coronary intervention
Source: Front Cardiovasc Med. 2026 Jan 12;12:1687231. doi: 10.3389/fcvm.2025.1687231 (PMC12832988; doi:10.3389/fcvm.2025.1687231)
Supplement: Supplementary file 1 [file Table1.docx]

Supplementary Table 1 Results of one-way Cox regression analyses for all-cause mortality

| Valuables | HR(95%CI) | *P* | Valuables | HR(95%CI) | *P* |
| --- | --- | --- | --- | --- | --- |
| Age |  |  | Previous MI |  |  |
| <45 | Reference |  | No | Reference |  |
| ≥65 | 2.59(1.84,3.65) | **<0.001** | Yes | 1.46(0.91,2.35) | 0.119 |
| Gender |  |  | Previous stroke |  |  |
| Male | Reference |  | No | Reference |  |
| Female | 1.03(0.77,1.39) | 0.831 | Yes | 1.08(0.68,1.70) | 0.745 |
| Diabetes |  |  | Smoking |  |  |
| No | Reference |  | No | Reference |  |
| Yes | 1.22(0.87,1.69) | 0.244 | Yes | 0.95(0.60,1.51) | 0.827 |
| Hypertension |  |  | Systolic pressure | 1.001(0.99,1.008) | 0.750 |
| No | Reference |  | Diastolic pressure | 1.003(0.99,1.02) | 0.684 |
| Yes | 0.99(0.73,1.37) | 0.989 | Total cholesterol | 0.95(0.83,1.09) | 0.485 |
| COPD |  |  | Hemoglobin | 0.99(0.98,1.00) | **0.193** |
| No | Reference |  | ALT | 1.002(1.001,1.003) | **<0.001** |
| Yes | 3.20(1.50,6.81) | **0.003** | AST | 1.002(1.001,1.003) | **<0.001** |
| Previous PCI |  |  | HDL-C | 1.13(0.59,2.14) | 0.712 |
| No | Reference |  | LDL-C | 1.02(0.85,1.22) | 0.829 |
| Yes | 1.22(0.81,1.84) | 0.342 |  |  |  |

HR：Hazards Ratio

Supplementary Table 2 Results of one-way Cox regression analysis of cardiac deaths

| Valuables | HR(95%CI) | *P* | Valuables | HR(95%CI) | *P* |
| --- | --- | --- | --- | --- | --- |
| Age |  |  | Previous MI |  |  |
| <45 | Reference |  | No | Reference |  |
| ≥65 | 2.24(1.26,3.96) | **0.006** | Yes | 0.64(0.20,2.06) | 0.458 |
| Gender |  |  | Previous stroke |  |  |
| Male | Reference |  | No | Reference |  |
| Female | 0.93(0.55,1.55) | 0.768 | Yes | 0.74(0.30,1.85) | 0.517 |
| Diabetes |  |  | Smoking |  |  |
| No | Reference |  | No | Reference |  |
| Yes | 0.92(0.50,1.70) | 0.783 | Yes | 0.69(0.28,1.72) | 0.422 |
| Hypertension |  |  | Systolic pressure | 1.00(0.99,1.02) | 0.407 |
| No | Reference |  | Diastolic pressure | 1.01(0.99,1.04) | 0.180 |
| Yes | 0.65(0.39,1.09) | 0.101 | Total cholesterol |  |  |
| COPD |  |  | Hemoglobin | 0.99(0.98,1.00) | 0.310 |
| No | Reference |  | ALT | 1.00(0.99,1.00) | 0.962 |
| Yes | 4.15(1.30,13.24) | **0.016** | AST | 1.00(0.99,1.00) | 0.543 |
| Previous PCI |  |  | HDL-C | 1.77(0.61,5.11) | 0.295 |
| No | Reference |  | LDL-C | 1.24(0.93,1.66) | 0.148 |
| Yes | 0.91(0.41,1.99) | 0.808 |  |  |  |

HR：Hazards Ratio

Supplementary Table 3 Subgroup analysis of the effect of different levels of TyG index on all-cause mortality

| Valuables | 6.08≤TyG<8.20 | 8.20≤TyG＜9.24 | 9.24≤TyG≤12.65 |
| --- | --- | --- | --- |
|  | HR (95%CI) | HR (95%CI) | HR (95%CI) |
| Gender |  |  |  |
| Male | 1.68(0.99,2.86) | Reference | 1.12(0.72,1.74) |
| Female | 2.04(0.79,5.30) | Reference | 1.95(1.20,3.15) * |
| Hypertension |  |  |  |
| No | 1.52(0.71,3.26) | Reference | 1.19(0.66,2.15) |
| Yes | 1.81(1.00,3.26) * | Reference | 1.53(1.04,2.24) * |
| Diabetes |  |  |  |
| No | 1.67(1.01,2.75) * | Reference | 1.46(0.99,2.14) |
| Yes | 3.03(0.86,10.69) | Reference | 1.10(0.57,2.14) |
| Previous MI |  |  |  |
| No | 1.98(1.23,3.17) * | Reference | 1.37(0.97,1.92) |
| Yes | 0.66(0.08,5.22) | Reference | 1.72(0.67,4.46) |
| Previous PCI |  |  |  |
| No | 1.95(1.20,3.15) * | Reference | 1.40(0.99,1.98) |
| Yes | 1.27(0.29,5.61) | Reference | 1.21(0.52,2.81) |
| Previous stroke |  |  |  |
| No | 1.99(1.24,3.19) * | Reference | 1.38(0.98,1.95) |
| Yes | 0.92(0.12,7.29) | Reference | 1.55(0.62,3.88) |

*Indicates P value < 0.05, the difference is significant.

Supplementary Table 4 Subgroup Analysis of the Effect of Different Levels of TyG Index on Cardiac Mortality

| Valuables | 6.08≤TyG<8.20 | 8.20≤TyG＜9.24 | 9.24≤TyG≤12.65 |
| --- | --- | --- | --- |
|  | HR (95%CI) | HR (95%CI) | HR (95%CI) |
| Gender |  |  |  |
| Male | 3.03(1.31,6.98) * | Reference | 1.43(0.67,3.09) |
| Female | 2.26(0.49,10.34) | Reference | 1.44(0.62,3.35) |
| Hypertension |  |  |  |
| No | 1.86(0.67,5.18) | Reference | 0.85(0.33,2.23) |
| Yes | 3.51(1.31,9.36) * | Reference | 2.03(0.97,4.27) |
| Diabetes |  |  |  |
| No | 2.31(1.06,5.02) * | Reference | 1.78(0.93,3.40) |
| Yes | 5.71(1.04,31.746)* | Reference | 0.74(0.21,2.55) |
| Previous PCI |  |  |  |
| No | 2.73(1.29,5.77) * | Reference | 1.49(0.82,2.72) |
| Yes | 2.75(0.30,25.57) | Reference | 0.54(0.09,3.32) |

*Indicates P value < 0.05, the difference is significant.
